# Effects of restrictive-prescribing stewardship on antibiotic consumption in primary care in China: an interrupted time series analysis, 2012–2017

**DOI:** 10.1186/s13756-020-00821-7

**Published:** 2020-09-25

**Authors:** Xuemei Wang, Yuqing Tang, Chenxi Liu, Junjie Liu, Youwen Cui, Xinping Zhang

**Affiliations:** 1 School of Medicine and Health Management, Tongji Medical College, Huazhong University of Science and Technology, Wuhan, Hubei Province China; 2 School of Statistics and Mathematics, Central University of Finance and Economics, Beijing, China

**Keywords:** Antibiotic consumption, Restrictive-prescribing stewardship, Primary care, Quality indictors of European surveillance of antimicrobial consumption (ESAC QIs), Interrupted time series analysis

## Abstract

**Background:**

The overuse of antibiotics has been a major public health problem worldwide, especially in low- and middle- income countries (LMIC). However, there are few policies specific to antibiotic stewardship in primary care and their effectiveness are still unclear. A restrictive-prescribing stewardship targeting antibiotic use in primary care has been implemented since December 2014 in Hubei Province, China. This study aimed to evaluate the effects of the restrictive-prescribing stewardship on antibiotic consumption in primary care so as to provide evidence-based suggestions for prudent use of antibiotics.

**Methods:**

Monthly antibiotic consumption data were extracted from Hubei Medical Procurement Administrative Agency (HMPA) system from Sept 1, 2012, to Aug 31, 2017. Quality Indictors of European Surveillance of Antimicrobial Consumption (ESAC QIs) combined with Anatomical Therapeutic Chemical (ATC) classification codes and DDD per 1000 inhabitants per day (DID) methodology were applied to measure antibiotic consumption. An interrupted time series analysis was performed to evaluate the effects of restrictive-prescribing stewardship on antibiotic consumption.

**Results:**

Over the entire study period, a significant reduction (32.58% decrease) was observed in total antibiotic consumption, which declined immediately after intervention (coefficient = − 2.4518, *P* = 0.005) and showed a downward trend (coefficient = − 0.1193, *P* = 0.017). Specifically, the use of penicillins, cephalosporins and macrolides/lincosamides/streptogramins showed declined trends after intervention (coefficient = − 0.0553, *P* = 0.035; coefficient = − 0.0294, *P* = 0.037; coefficient = − 0.0182, *P* = 0.003, respectively). An immediate decline was also found in the contribution of β-lactamase-sensitive penicillins to total antibiotic use (coefficient = − 2.9126, *P* = 0.001). However, an immediate increase in the contribution of third and fourth-generation cephalosporins (coefficient = 5.0352, *P* = 0.005) and an ascending trend in the contribution of fluoroquinolones (coefficient = 0.0406, *P* = 0.037) were observed after intervention. The stewardship led to an immediate increase in the ratio between broad- and narrow-spectrum antibiotic use (coefficient = 1.8747, *P* = 0.001) though they both had a significant downward trend (coefficient = − 0.0423, *P* = 0.017; coefficient = − 0.0223, *P* = 0.006, respectively). An immediate decline (coefficient = − 1.9292, *P* = 0.002) and a downward trend (coefficient = − 0.0815, *P* = 0.018) were also found in the oral antibiotic use after intervention, but no significant changes were observed in the parenteral antibiotic use.

**Conclusions:**

Restrictive-prescribing stewardship in primary care was effective in reducing total antibiotic consumption, especially the use of penicillins, cephalosporins and macrolides/lincosamides/streptogramins. However, the intervention effects were limited regarding the use of combinations of penicillins with ß-lactamase inhibitors, the third and fourth-generation cephalosporins, fluoroquinolones and parenteral antibiotics. Stronger administrative regulations focusing on specific targeted antibiotics, especially the use of broad-spectrum antibiotics and parenteral antibiotics, are in urgent need in the future.

## Introduction

Antimicrobial resistance (AMR) has been increasingly concerned as a major public health problem worldwide, leading to longer hospital stays, higher medical costs and increased mortality [1, 2]. It was estimated that approximately 25,000 deaths in Europe, and 23,000 deaths in the USA were caused by AMR each year [3]. The health care costs resulted from antibiotic-resistant infections were as high as $20 billion and the lost productivity was estimated to be $35 billion per year in the US [4]. If no actions are taken, up to 10 million additional lives would be lost and the total economic burden would reach $100 trillion by 2050 [5].

The intensive use of antibiotics has been regarded as a main driver of AMR and the unnecessary use further worsened such situation [1, 6]. The global antibiotic consumption increased by 65% between 2000 and 2015, which was primarily driven by increased consumption in low- and middle-income countries (LMICs) [7]. It was reported that appropriately 50% of patients were prescribed antibiotics in healthcare institutions, with 30–50% regarded unnecessary and/or inappropriate [8]. It has been widely recognized that primary care should take main responsibilities because the most majority of antibiotic consumption tend to occur in primary care, which has been demonstrated by the fact that approximately half of patients attending primary care received at least one antibiotic in LMICs [9].

There are few policies specific to antibiotic stewardship in primary care and little is known about the practical effectiveness. Although previous studies have conducted some small-sale interventions to regulate antibiotic overuse in primary care, but the effects are mixed and confused. As examples, online evidence-based feedbacks effectively reduced antibiotic prescribing from 37.4 to 28.1% in patients with suspected respiratory tract infections (RTIs) attending primary care [10]. Similarly, a weekly prospective audit and feedback in three community facilities brought about a 25% immediate reduction in all antibiotic prescriptions after conducting the antibiotic stewardship and a continuous 5% decrease over the entire intervention [11]. However, other studies showed inconsistent findings. A systematic review summarizing educational interventions to improve antibiotic prescribing showed that 62% of studies in primary care reported positive results for all measured outcomes, including total antibiotic prescription, prescribing attitudes and behaviors, and etc.; 30% reported partial results that were not statistically significant and the remaining studies failed to report any significant improvements [12]. Further high-level evidence is needed to instruct intervention strategies in regulating antibiotic consumption in primary care.

Previous antibiotic stewardship programs in China mainly focus on secondary and tertiary hospitals, which have achieved significant improvements [13]. However, very few detailed or targeted policies are specific to antibiotic consumption in primary care, leading to a relatively poor regulation efficiency on them. For instance, an increasing trend was observed in the overall antibiotic consumption in urban primary healthcare centers in Shandong province of China, revealing an urgent need for strengthened regulation on antibiotic use in primary care [14]. Therefore, it is necessary to evaluate the intervention effects on antibiotic use in primary care, especially on some specific antibiotics, so as to supplement more targeted evidence.

A restrictive-prescribing stewardship with specified administrative regulation was issued in 2014 in Hubei province of China, aiming at further promoting prudent use of antibiotics [15]. This administrative regulation put forward detailed requirements specific to antibiotic prescribing in primary care, featured as concretely restricting antibiotic grades, varieties, prescribing rate and route of administration. This study aimed to evaluate the effects of this restrictive-prescribing stewardship on antibiotic consumption in primary care, especially with the advantage of an internationally comparable methodology of Quality Indictors of European Surveillance of Antimicrobial Consumption (ESAC QIs) [16]. The findings will fill the gaps in literature, especially supplementing comparable evidence on the antibiotic stewardship in primary care, so as to further promote prudent use of antibiotics.

## Methods

### Settings and data sources

This study was conducted in Hubei province, central China. Hubei has a population of over 61 million and covers an area of 185,900 km^2^. Its per capita gross domestic product (GDP) is 60,198.68 yuan ($8915.95 USD), ranking in the middle range of all provinces. The disposable income for rural residents and urban residents is 13,812.09 yuan ($2059.38 USD) and 31,889.42 yuan ($4754.71 USD) respectively (2017). There are 36,323 healthcare institutions in Hubei, among which 34,742 are primary care institutions, including state-owned community or township centers [17].

Data used in this study were extracted from the Hubei Medical Procurement Administrative Agency (HMPA) system, which provided a specialized and reliable source on the medicine consumption, including antibiotics [18]. The database is targeted for primary care institutions, and monthly procurement data are recorded and updated (e.g. medicine name, procurement volume, dosage form). According to the requirements of HMPA, primary care institutions are allowed to stock and dispense medicines listed in the Essential Medicines List (EML) from its procurement platform, except for few supplementary medicines, e.g. emergency medicine. All the valid procurement records of antibiotics between September 2012 and August 2017 (except for few blank procurement records) were collected for our study.

### Description of the restrictive intervention

In November 2014, a restrictive-prescribing stewardship specific to antibiotics was developed and has been formally implemented since December 2014 across the entire Hubei province. This restrictive intervention put forward strict administrative rules on the antibiotic grades, varieties, prescribing rate and route of administration in primary care. Four major requirements were involved as follows.

Firstly, all primary care institutions should comply with the requirements on using non-restricted and restricted antibiotics; only physicians with corresponding prescribing privileges are qualified to prescribe relevant grades of antibiotics; Secondly, the number of antibiotic varieties should be strictly controlled within 30 in primary care and 15 for village clinics; Thirdly, the prescribing rate of antibiotics should not exceed 20% for outpatients in rural township health centers, 40% for emergency patient, and 60% for inpatients; the Antibiotics Use Density (AUD) should not exceed 40 DDDs per 1000 inhabitants per day, and etc.; Fourthly, the route of administration of antibiotics in primary care should be mainly oral or intramuscular injection; intravenous infusion or intravenous bolus injection should be restricted to inpatient departments or outpatient observation rooms; intravenous infusion in clinics (e.g. village clinics, urban community health stations) should be approved by local health bureau.

Under the administrative power, a good policy implementation was guaranteed. Specifically, local healthcare institutions strictly execute the above four restrictive regulations by establishing and implementing antibiotic classification management system, strengthening the monitoring and management of clinical use, and enhancing the training and assessment on prescribers. Physicians follow the principles of safety, effectiveness and economy to prescribe antibiotics rationally, and only those who have received adequate training and passed assessment are granted with corresponding antibiotic prescribing privileges. Local health bureau as well as provincial health bureau are responsible for managing, supervising and evaluating the use of antibiotics. Additionally, performance appraisal are linked with rewards (e.g. salary, title appraisal), and penalties are also executed for violations of the regulations [15].

### Measurement of antibiotic consumption

The Anatomical Therapeutic Chemical (ATC) classification codes were used to categorize medicines, and procurement records of J01 (antibiotics for systemic use) were extracted for the purpose of this study [19]. Defined daily dose (DDD) was used to measure the volume of antibiotics according to the WHO Collaborating Centre for Drug Statistics Methodology [20]. DDD equivalence per package (DPP) was expressed in DDD units [(unit strength × pack size)/DDD]. The summed DPPs of all-inclusive products formed the total volume for each group of antibiotic consumption (DDDs). To strengthen the comparability of antibiotic use, the DDD per 1000 inhabitants per day (DID) was eventually transformed to calculate antibiotic consumption as DID provides a measure of exposure or therapeutic intensity in a defined population, allowing comparisons across various time periods and population groups [21]. The figure “10 DID” can be explained as: 10 DDDs of the drug are averagely utilized in a representative group of 1000 inhabitants, on any given day of the year analyzed; or expressed as 1% of the population are receiving this drug each day in that year [21].

To provide drug-specific insight in measuring antibiotic consumption and trigger action to improve antibiotic use, quality indictors (QIs) proposed by European Surveillance of Antimicrobial Consumption (ESAC) were used to measure antibiotic use, which has become an internationally comparable methodology [16, 22]. In this study, 10 ESAC QIs were selected into analysis and the remaining 2 ESAC QIs were not included because they were not suitable for the statistical methodology in this study. Of note, the use of narrow-spectrum antibiotics and broad-spectrum antibiotics were key indicators among ESAC QIs that deserved more attention. The narrow-spectrum antibiotics refer to the antibiotic agents that act against a limited range of potential pathogens and are often used for the specific infection when the causative agent of the infection is known, whereas broad-spectrum antibiotics are those act against a wider range of pathogenic bacteria and are more likely to kill both potential pathogens and normal flora [23–26]. Generally, narrow-spectrum antibiotics are regarded more ideal than broad-spectrum counterparts [25]. In this study, when calculating the ratio between broad- and narrow-spectrum antibiotic use, combinations of penicillins with ß-lactamase inhibitors, second generation cephalosporins, third generation cephalosporins and macrolides/lincosamides/streptogramins (minus erythromycin) correspond to broad-spectrum antibiotics, whereas ß-lactamse-sentitive penicillins, first generation cephalosporins and erythromycin correspond to narrow-spectrum antibiotics [16, 22]. In addition, 4 local QIs were developed after considering the policy context in China (Table 1).

**Table 1:** Quality indicators of measuring antibiotic consumption in this study

NO.	Quality indicators
ESAC QIs 1–5 are the use of relevant antibiotics (expressed in DID):	
*1*	*total use of systemic antibiotics (J01)*
*2*	*use of penicillins (J01C)*
*3*	*use of cephalosporins (J01D)*
*4*	*use of macrolides/lincosamides/streptogramins (J01F)*
*5*	*use of quinolones (J01M).*
ESAC QIs 6–9 are the relative contributions (% of total J01 use) of:	
*6*	*ß-lactamse-sentitive penicillins (J01CE)*
*7*	*combinations of penicillins with ß-lactamase inhibitors (J01CR)*
*8*	*third and fourth generation cephalosporins (J01DD/DE)*
*9*	*fluoroquinolones (J01MA)*
ESAC QI 10 is the ratio between broad- and narrow-spectrum antibiotics:	
*10*	*(J01CR + J01DC + J01DD + J01F (minus J01FA01)/(J01CE + J01DB + J01FA01)*
QIs 11–14 are adapted according to the policy context in China (expressed in DID):	
*11*	*use of broad-spectrum antibiotics*
*12*	*use of narrow-spectrum antibiotics*
*13*	*use of oral antibiotics*
*14*	*use of parenteral antibiotics*

### Statistical analysis

Regular, evenly spaced intervals are appropriate for a segmented regression analysis to evaluate the effect of intervention [27]. In this research, monthly antibiotic consumption from Sept 1, 2012, to Aug 31, 2017 was applied as analytical unites, of which the period from Sept 1, 2012 to December 2014 was pre-intervention evaluation period and from January, 2015 to Aug 31, 2017 was post-intervention evaluation period.

To estimate the effect of restrictive prescribing stewardship on antibiotic use, the following segmented linear regression model was applied [28]:

$$ {Y}_t={\beta}_0+{\beta}_1\ast {Time}_t+{\beta}_2\ast {Intervention}_t+{\beta}_3\ast Time\ {after\ intervention}_t+{\beta}_4\ast \sin \left(2\Pi {Time}_t/12\right)+{\beta}_5\ast \cos \left(2\Pi {Time}_t/12\right)\ {\varepsilon}_t $$

As two key parameters in the segmented linear regression, level and trend define each segment of a time series. The level is the value of the series at the beginning of a given time interval, while the trend is the rate of change of the intervention measure.

Here, *Y*_*t*_ is the average number of antibiotic use in month t; Time is a continuous variable indicating time in months at time t from the start of observation; intervention is an indicator for *Time*_*t*_ occurring before (intervention = 0) or after (intervention = 1) the cap, which was implemented at month 28 (December, 2014) in the series; and *Time after intervention*_*t*_ is a continuous variable counting the number of months after the intervention at time t, coded 0 before the cap and added by 1 continuously after the cap. The coefficients *β*_0_ and *β*_1_ respectively estimate the baseline level of outcome at time zero, and the change that occurs with each month before the intervention; *β*_2_ and *β*_3_ respectively estimate the level change in the average monthly number of antibiotic use immediately after the intervention, and the trend change of indicators after the cap, compared with the monthly trend before the cap; The sum of *β*_1_ + *β*_3_ is the post intervention slope. *β*_4_ and *β*_5_ were used to adjust for a potential seasonality effect [29]. The error term *ε*_*t*_ represents the random variable not explained by the model at time t. Considering autocorrelation may underestimate standard errors and overestimate significance of the intervention effects, it is necessary to correct for the possibility of autocorrelation [30]. The most popular test to ascertain the presence or absence of autocorrelation is Durbin-Watson test, which is a number that tests for autocorrelation in the residuals, ranging from 0 to 4 [31–33]. Values approaching 2 means that there is almost no autocorrection, whereas approaching 0 indicate positive autocorrection and approaching 4 indicate negative autocorrection [33]. Therefore, Durbin-Watson test was used to check for autocorrelation in this study.

All statistical analyses above were performed using STATA version 12.0 (STATA Corp, College Station, TX, USA) and *P* < 0.05 was considered statistically significant.

## Results

### Overall antibiotic consumption over the entire study period

**Fig. 1:**
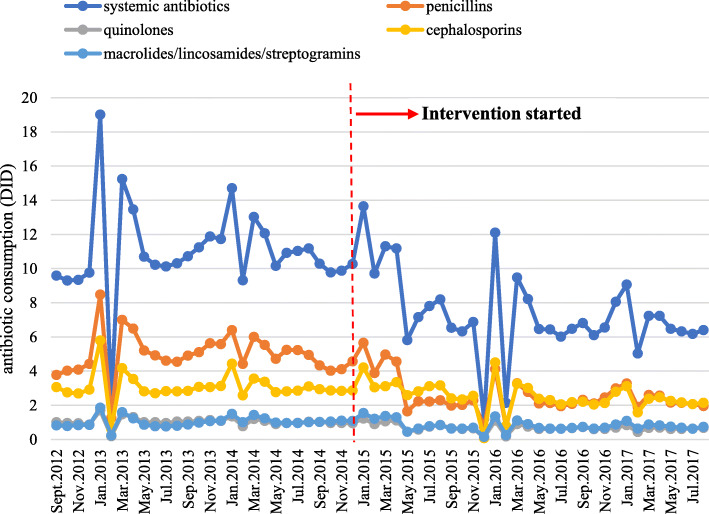
Monthly antibiotic consumption of systemic antibiotics, penicillins, quinolones, cephalosporins and macrolides/lincosamides/streptogramins

Over the entire study period, the total antibiotic consumption in primary care in Hubei province declined from 11.02 DID to 7.43 DID (32.58% decrease). For penicillins and cephalosporins, the consumption respectively decreased from 5.01 DID to 2.64 DID (47.31% decrease) and 3.08 DID to 2.54 DID (17.53% decrease). For quinolones and macrolides/lincosamides/streptogramines, the consumption declined from 1.05 DID to 0.71 DID (32.38% decrease) and 1.03 DID to 0.82 DID (20.39% decrease) respectively (Fig. 1).

**Fig. 2:**
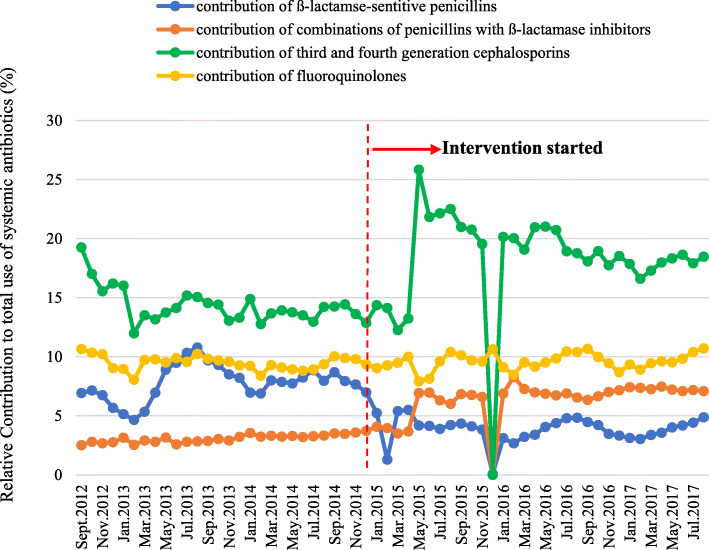
Monthly relative contributions of ß-lactamse-sentitive penicillins, combinations of penicillins with ß-lactamase inhibitors, third and fourth generation cephalosporins, fluoroquinolones to total use of systemic antibiotics

The relative contributions of ß-lactamse-sentitive penicillins to total antibiotic use declined from 7.78 to 4.12% (47.04% decrease). However, the relative contributions of penicillins with ß-lactamase inhibitors, third and fourth generation cephalosporins and fluoroquinolones to total antibiotic use respectively increased from 3.08 to 6.19%, 14.43 to 18.13%, and 9.51 to 9.56% (100.97% increase, 25.64% increase, 0.53% increase, respectively) (Fig. 2).

**Fig. 3:**
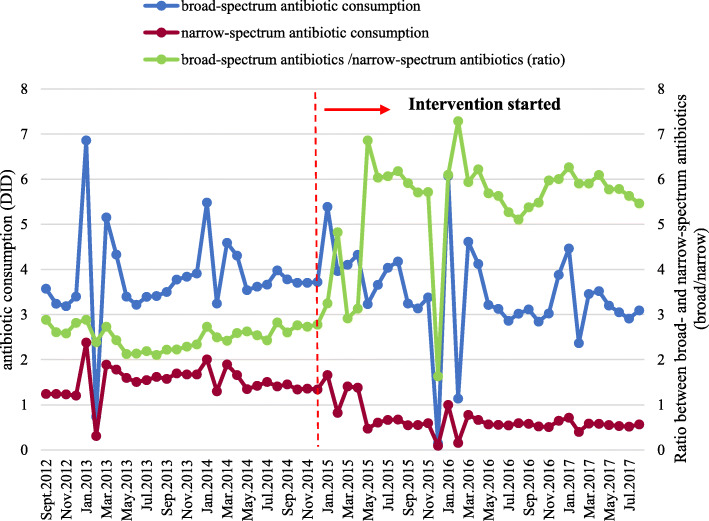
Monthly broad- and narrow-spectrum antibiotic consumption as well as the ratio between them

The consumption of broad-spectrum antibiotics and narrow-spectrum antibiotics respectively declined from 3.82 DID to 3.20 DID (16.23% decrease) and 1.51 DID to 0.68 DID (54.97% decrease). However, the ratio between broad- and narrow-spectrum antibiotic consumption increased from 2.51 to 5.09 (Fig. 3).

**Fig. 4:**
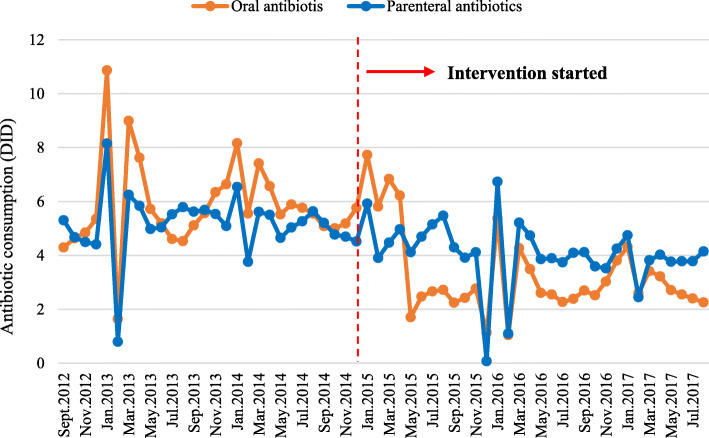
Monthly antibiotic consumption of oral and parenteral antibiotics

The consumption of oral antibiotics and parenteral antibiotics respectively declined from 5.84 DID to 3.33 DID (42.98% decrease), and 5.18 DID to 4.09 DID (21.04% decrease). The average decline of oral antibiotic consumption was greater than that of parenteral antibiotics over the entire study period (Fig. 4).

### Effects of restrictive-prescribing stewardship on antibiotic consumption

As shown in Table 2, all of the Durbin-Watson statistics were approaching 2, indicating that there was no autocorrelation in the observations. Before implementing the restrictive-prescribing stewardship, the consumption of total antibiotics showed an ascending trend that was not statistically significant (coefficient = 0.0237, *P* = 0.554), while the consumption declined immediately after intervention (coefficient = − 2.4518, *P* = 0.005) and had a significant downward trend (coefficient = − 0.1193, *P* = 0.017). The consumption of penicillins, macrolides/lincosamides/streptogramins, quinolones declined immediately after intervention (coefficient = − 1.9109, *P* < 0.001; coefficient = − 0.2248, *P* = 0.030; coefficient = − 0.2019, *P* = 0.019, respectively), and the consumption of penicillins, cephalosporins and macrolides/lincosamides/streptogramins showed declined trends after intervention (coefficient = − 0.0553, *P* = 0.035; coefficient = − 0.0294, *P* = 0.037; coefficient = − 0.0182, *P* = 0.003, respectively).

**Table 2:** The segmented regression analysis of intervention on antibiotic consumption

	Baseline level (95% CI)	Baseline trend (95% CI)	Level change after intervention (95% CI)	Trend change after intervention (95% CI)	DW
***Use of relevant antibiotics (expressed in DID)***					
Systemic antibiotics	10.7777*** (9.51, 12.05)	0.0237 (− 0.06, 0.10)	− 2.4518** (− 4.14, − 0.76)	− 0.1193* (− 0.22, − 0.02)	2.0572
Penicillins	4.8920*** (4.22, 5.56)	0.0124 (− 0.03, 0.05)	− 1.9109*** (− 2.80, − 1.02)	−0.0553* (− 0.11, − 0.00)	1.9703
Cephalosporins	3.1150*** (2.75, 3.48)	− 0.0015 (− 0.02, 0.02)	− 0.0262 (− 0.51,0.45)	−0.0294* (− 0.06, − 0.00)	2.2194
Macrolides/lincosamides/ streptogramins	0.9053*** (0.75, 1.06)	0.0099* (0.00, 0.02)	− 0.2248* (− 0.43, − 0.02)	− 0.0182** (− 0.03, − 0.01)	1.9409
Quinolones	1.0594*** (0.93, 1.19)	−0.0005 (− 0.01, 0.01)	− 0.2019* (− 0.37, − 0.03)	− 0.0077 (− 0.02,0.00)	2.0270
***Relative contributions (% of total J01 use)***					
ß-lactamse-sentitive penicillins	7.2141*** (5.86, 8.57)	0.0271 (− 0.06, 0.11)	− 2.9126** (− 4.55, − 1.27)	− 0.0825 (− 0.19, 0.03)	2.0439
Combinations of penicillins with ß-lactamase inhibitors	2.6349*** (1.71, 3.56)	0.0312 (−0.03, 0.09)	1.2010 (− 0.01, 2.41)	0.0618 (− 0.01, 0.13)	1.9534
Third and fourth generation cephalosporins	16.2036*** (13.55, 18.85)	−0.1312 (− 0.30, 0.03)	5.0352** (1.55, 8.52)	0.1543 (− 0.05, 0.36)	1.9815
Fluoroquinolones	9.7988*** (9.31, 10.29)	−0.0246 (− 0.05, 0.01)	0.2156 (− 0.42, 0.85)	0.0406* (0.00, 0.08)	1.8880
***Use of Broad- and narrow-spectrum antibiotics (expressed in DID)***					
Broad	3.6089*** (3.16, 4.06)	0.0148 (−0.01, 0.04)	−0.1212 (− 0.72, 0.48)	−0.0423* (− 0.08, − 0.01)	2.2142
Narrow	1.4481*** (1.24, 1.65)	0.0055 (− 0.01, 0.02)	− 0.6449*** (− 0.92, − 0.37)	− 0.0223** (− 0.04, − 0.01)	1.9398
***Ratio between broad-narrow spectrum antibiotic use (ratio)***					
Broad /Narrow	2.4977*** (1.65, 3.34)	0.0032 (− 0.05, 0.06)	1.8747** (0.78, 2.97)	0.0500 (− 0.02, 0.12)	1.9345
***Use of oral and parenteral antibiotics (expressed in DID)***					
Oral	5.6076*** (4.73, 6.48)	0.0221 (− 0.03, 0.08)	−1.9292** (−3.09, − 0.77)	−0.0815* (− 0.15, − 0.01)	1.9424
Parenteral	5.1721*** (4.63, 5.71)	0.0011 (− 0.03, 0.03)	−0.5049 (− 1.22, 0.21)	−0.0373 (− 0.08, 0.00)	2.2052

Notes: *p < 0.05; **p < 0.01; ***p < 0.001;

*CI* Confidence intervals, *DW* Durbin-Watson

The restrictive-prescribing stewardship was associated with an immediate decline in the contribution of β-lactamase-sensitive penicillins to total antibiotic use (coefficient = − 2.9126, *P* = 0.001). However, an immediate increase was observed in the contribution of third and fourth-generation cephalosporins to total antibiotic use (coefficient = 5.0352, *P* = 0.005), and an ascending trend was found in the contribution of fluoroquinolones to total antibiotic use (coefficient = 0.0406, *P* = 0.037) after intervention.

The stewardship also led to an immediate increase in the ratio between broad- and narrow-spectrum antibiotic use (coefficient = 1.8747, *P* = 0.001) though they both had a significant downward trend (coefficient = − 0.0423, *P* = 0.017; coefficient = − 0.0223, *P* = 0.006, respectively).

Finally, the stewardship was associated with an immediate decline in the consumption of oral antibiotics (coefficient = − 1.9292, *P* = 0.002) and a continuous downward trend (coefficient = − 0.0815, *P* = 0.018). However, no significant changes were found in the consumption of parenteral antibiotics after intervention (Table 2).

## Discussion

### Summary of main findings

This study confirmed that the restrictive-prescribing stewardship achieved positive effects in declining total antibiotic use, which attained the major target of the intervention. However, the intervention effects on the consumption of specific antibiotics were mixed. Although a significant decline was observed in the use of penicillins, cephalosporins, macrolides/lincosamides/streptogramins, the intervention effects on combinations of penicillins with ß-lactamase inhibitors, the third and fourth-generation cephalosporins, fluoroquinolones and parenteral antibiotics were limited, which deserved more attention and discussion.

### Strengths and limitations of the study

To the best of our knowledge, it was the first study that attempted to evaluate the effects of restrictive-prescribing stewardship on antibiotic consumption in primary care, with the advantage of drug-specific quality indicators of ESAC (ESAC QIs) combined with an interrupted time series design and DID methodology.

There are several limitations in this study. First, not all antibiotics consumed in primary care institutions were included in this study. The data of non-prescribed antibiotic use (e.g. self-medication at home or over-the-counter) and prescriptions in private primary care facilities were not available in the present study. Second, the data used in this study were procurement data instead of directly extracting from the actual medicine use in each institution. However, the procurement data were based on the current practical consumption, which was approximately equivalent to the actual medicine use. Third, we are not able to assess the rationale of the cut-off value made by the government since the relevant process were not made public. Finally, clinical outcomes were not involved in this study as relevant data were not available. Future studies should focus on the safety of restrictive-prescribing stewardship (e.g. return visits, hospitalization rates and overall mortality) if clinical-related data are available.

### Comparison with existing literature

This study confirmed that the restrictive-prescribing stewardship was associated with a significant reduction in total antibiotic consumption, indicating that the stewardship achieved success to a large extent. As pointed out by Coenen S et al. [16], “total use of systemic antibiotics expressed in DID” was regarded as the most useful indicator as it could best represent the size of pressure driving AMR, thus the reduction in total antibiotic use in our study indicated a lessening pressure driving resistance. Contrast with our findings, Borde JP et al. [34] found that the total antibiotic use expressed in DID increased by 2% in Germany after implementing an intensified antibiotic stewardship (aiming at a > 30% reduction of cephalosporin and fluoroquinolone use within 1 year), which indicated that stewardship targeting only several antibiotics may be insufficient to decrease the total antibiotic use.

With regard to the use of penicillins, it was recommended that the use of cephalosporins, macrolides/lincosamides/streptogramins, quinolones should be assessed together [16]. Along with the declined use of penicillins after intervention, the use of cephalosporins, macrolides/lincosamides/streptogramins also achieved significant reduction, indicating relevant practice is getting better as the latter antibiotics acted as second-line drugs with limited evidence of additional clinical benefit over penicillins for the most common indication [16].

Of note, the contribution of β-lactamase-sensitive penicillins to total antibiotic use showed an immediate decline after intervention, while an increase was observed in the contribution of combinations of penicillins with ß-lactamase inhibitors though there was no statistical significance, which was similar with the phenomenon in European countries that a shift from the use of narrow-spectrum antibiotics towards combinations of penicillins with ß-lactamase inhibitors [35].

In addition, similar to the declined cephalosporin use in our study, Borde JP et al. [34] found that the use of cephalosporins expressed in DID declined by 26% after implementing an intensified antibiotic stewardship targeting cephalosporin and fluoroquinolone use, indicating interventions focusing on specific antibiotics would be more targeted and effective to reduce corresponding antibiotic use. However, an immediate increase was observed in the contribution of third and fourth-generation cephalosporins in our study, indicating a relatively poor practice. One reason may lie in that third-generation cephalosporins were frequently prescribed for common infections by general physicians, while the prescribing habits were usually difficult to change [36]. Another reason may because the third and fourth-generation cephalosporins could be generally used when patients had immunoglobulin E-mediated response (anaphylaxis) to penicillin, which may also result in an increase of third and fourth-generation cephalosporin use [37].

Regarding the use of quinolones, it was worth noting that fluoroquinolone was the only quinolone product used in primary care of Hubei Province. In this study, though fluoroquinolone use showed an absolute decrease, the subsequent downward trend was nonsignificant, and an ascending trend was found in the contribution of fluoroquinolones, illustrating that the regulatory control on fluoroquinolone use need to be further strengthened. Consistent with our findings, Rodrigues AT et al. [38] found that relative prescription of fluoroquinolones showed no changes after intervention, which were attributed to the association of these prescriptions in primary care with disease condition and patient’s age, making it more difficult to change prescribing behaviors in such cases. Jindai K et al. [39] discovered an initial reduction on fluoroquinolone prescribing rate after implementing fluoroquinolone safety initiative but was not sustained later, calling for sustainable strategies. Future stewardship should not only specifically target fluoroquinolone use but also combine ongoing approaches to enhance the sustainability of intervention.

Another interesting finding in this study was that along with the reduction of total antibiotic use, an increase of the ratio between broad- and narrow-spectrum antibiotic use was observed after intervention, though there was a significant downward trend respectively in the use of broad- and narrow-spectrum antibiotics. This finding was consistent with a previous study by Li H et al. [40] that the percentage of broad-spectrum antibiotic prescriptions did not achieve significant decline after intervention, which were attributed to less attention on the appropriateness of antibiotic types. Based on the comparison with other previous studies [38, 41–46], the increase of the ratio between broad- and narrow-spectrum antibiotic use in this study may result from the following reasons. First, our restrictive-prescribing stewardship lacked multi-faceted intervention (e.g. a multidisciplinary approach involving physicians, pharmacists and patients simultaneously) and was not targeted on some specific excessively-used broad-spectrum antibiotics. Second, our stewardship neglected advocating guideline-recommended alternative antibiotics, especially relevant narrow-spectrum alternatives, to replace those excessively-used broad-spectrum antibiotics. Third, the consumption of broad-spectrum antibiotics has always occupied a major proportion of total antibiotics and presented a dramatical increase, making it more difficult to decline the ratio between broad- and narrow-spectrum antibiotic use. Fourth, broad-spectrum antibiotics are usually inappropriately preferred even in the instances that narrow-spectrum counterparts maybe more suitable, and physicians tend to broaden empiric antibacterial therapy especially when facing patients with worsening condition. Finally, in some cases (e.g. neutropenic sepsis), physicians generally tried empiric broad-spectrum therapy at the beginning before the microbiological test results were known, which may also increase the difficulty of reducing broad-spectrum antibiotic use.

Moreover, the stewardship failed to make significant progress in regulating parenteral antibiotic use, though significant decline were found in oral antibiotic use. Contrast with our findings, Hersh AL et al. [47] found a decline in parenteral antibiotic use after implementing an outpatient parenteral antibiotic therapy (OPAT) stewardship program, which may attribute to the more targeted stewardship specific to parenteral antibiotics. The overuse of injections has become common concerns in many LMICs and may result in unexpected outcomes [48]. However, a wide belief still existed in many cultures that injection is a quite powerful method for health, driving patients preferring injections to oral medicines [49]. Not only that, physicians are more likely to prescribe injections to satisfy patients’ demands and earn a higher salary due to their higher prices [49].

### Policy implications

It is a well-known fact that the overuse of antibiotics is increasingly regarded as a main pressure driving resistance, resulting in a cost to human lives and healthcare resources [1, 2]. Restrictive-prescribing stewardship successfully regulated the excessive use of antibiotics overall. However, regarding the use of combinations of penicillins with ß-lactamase inhibitors, the third and fourth-generation cephalosporins, fluoroquinolones and parenteral antibiotics, a stronger and more targeted antibiotic stewardship involving multidisciplinary approaches is needed. Future stewardship focusing on targeted antibiotics especially the use of broad-spectrum antibiotics and parenteral antibiotics, combined with multi-faceted ongoing interventions are warranted to achieve sustainable improvement.

## Conclusion

Restrictive-prescribing stewardship in primary care achieved significant effects in reducing total antibiotic consumption, indicating a major success of the intervention to a large extent. Practice regarding the use of penicillins, cephalosporins, macrolides/lincosamides/streptogramins are getting better overall, whereas the intervention effects on combinations of penicillins with ß-lactamase inhibitors, the third and fourth-generation cephalosporins, fluoroquinolones and parenteral antibiotics were limited. Stronger regulation with a multidisciplinary approach should be strengthened to focus on the use of targeted antibiotics, especially broad-spectrum antibiotics and parenteral antibiotics. Future studies are warranted to explore the potential influencing factors of limited effects on those antibiotics and accordingly design a stronger and more targeted antibiotic stewardship strategy.

## Data Availability

The original data used in this study were extracted from the Hubei Medical Procurement Administrative System (HMPAS), which is a publicly available platform.

## Abbreviations

ATC: Anatomical Therapeutic Chemical classification
DID: DDD per 1000 inhabitants per day
ESAC QIs: Quality Indictors of European Surveillance of Antimicrobial Consumption
J01: Antibiotics for systemic use
J01C: Penicillins
J01D: Cephalosporins
J01F: Macrolides/lincosamides/streptogramins
J01M: Quinolones
J01CE: ß-lactamse-sentitive penicillins
J01CR: Combinations of penicillins with ß-lactamase inhibitors
J01DD/DE: Third and fourth generation cephalosporins
J01MA: Fluoroquinolones
J01DC: Second generation cephalosporins
J01FA01: Erythromycin
J01DB: First generation cephalosporins
CI: Confidence intervals
DW: Durbin-Watson
